# Supplementary material for: Physico-Chemical and Sensory Characteristics of Extruded Cereal Composite Flour Porridge Enriched with House Crickets (Acheta domesticus)
Source: Foods. 2025 Aug 20;14(16):2893. doi: 10.3390/foods14162893 (PMC12385631; doi:10.3390/foods14162893)
Supplement: Supplementary file 1 [file foods-14-02893-s001.zip › Questionnaire S1.pdf]

Supplementary Questionnaire S1: Acceptance testing sensory ballot cricket-enriched porridge products

Gender:……………………………………..Date:…………………..Panelist No:…………….

Please rinse your mouth with water before starting and in-between different samples.

You are provided with two porridge samples coded 101 and 601. Please taste each product in the order presented from left to right; and rank your degree of liking for each attribute and general acceptability using the 9-point hedonic scale provided below. You may taste as much as you may like but you must finish at least one third of the sample.

Rating scale

**9**	**8**	**7**	**6**	**5**	**4**	**3**	**2**	**1**
Like extremely	Like very much	Like moderately	Like slightly	Neither like nor dislike	Dislike slightly	Dislike moderately	Dislike very much	Dislike extremely

**Product code**	**Appearance**	**Texture**	**Color**	**Mouth feel**	**Taste**	**Aroma**	**General acceptability**
101							
601							

General comments

……

Which of the two (2) porridges would you prefer?

……

What do you like most about the porridge you prefer?

……

What don't you like/would like to be changed about the porridge you preferred?

……
