# Supplementary figures and images for: Physico-Chemical and Sensory Characteristics of Extruded Cereal Composite Flour Porridge Enriched with House Crickets (Acheta domesticus)
Source: Foods. 2025 Aug 20;14(16):2893. doi: 10.3390/foods14162893 (PMC12385631; doi:10.3390/foods14162893)

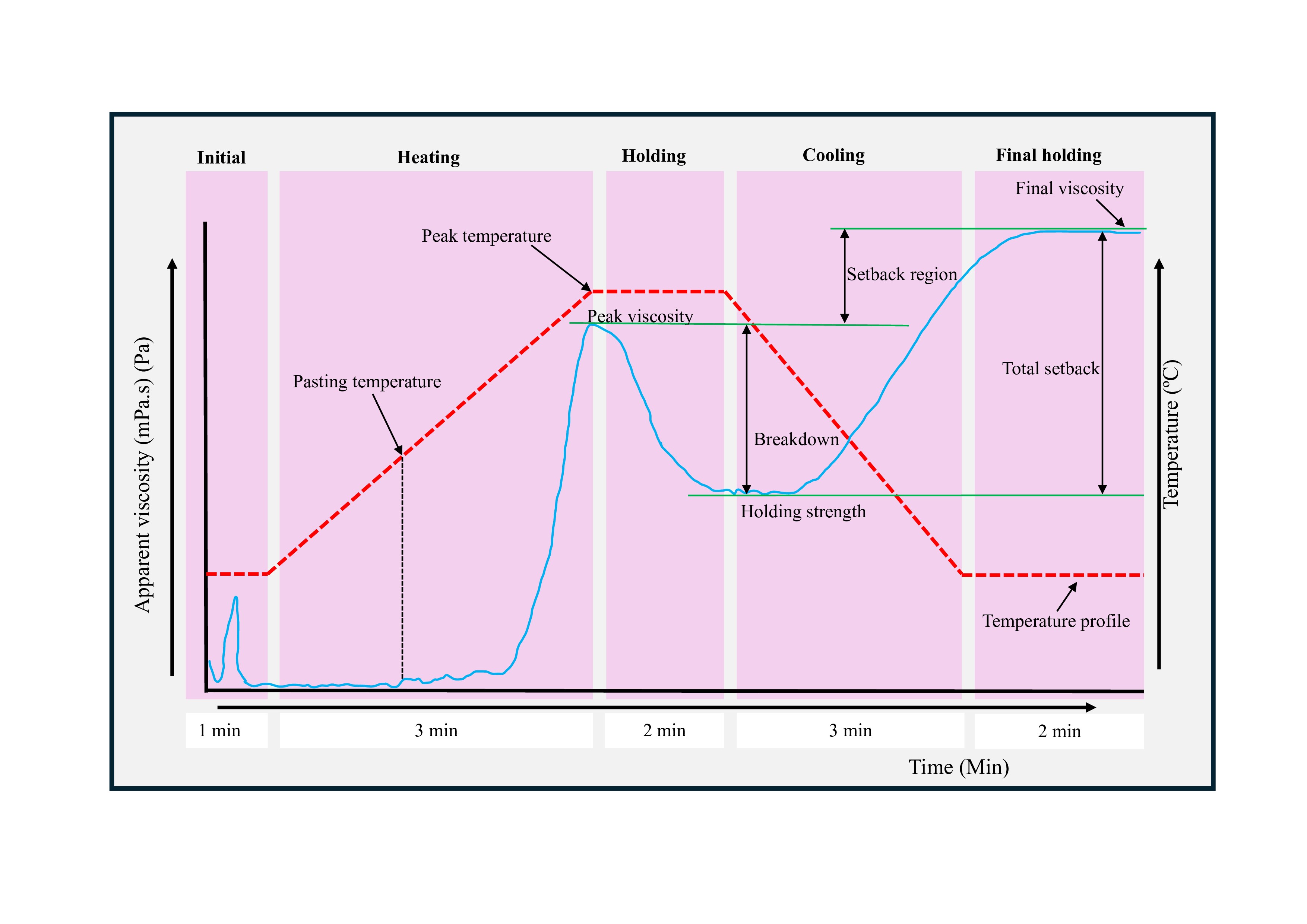

Supplement: Supplementary file 1 [file foods-14-02893-s001.zip › Fig S1.jpg]

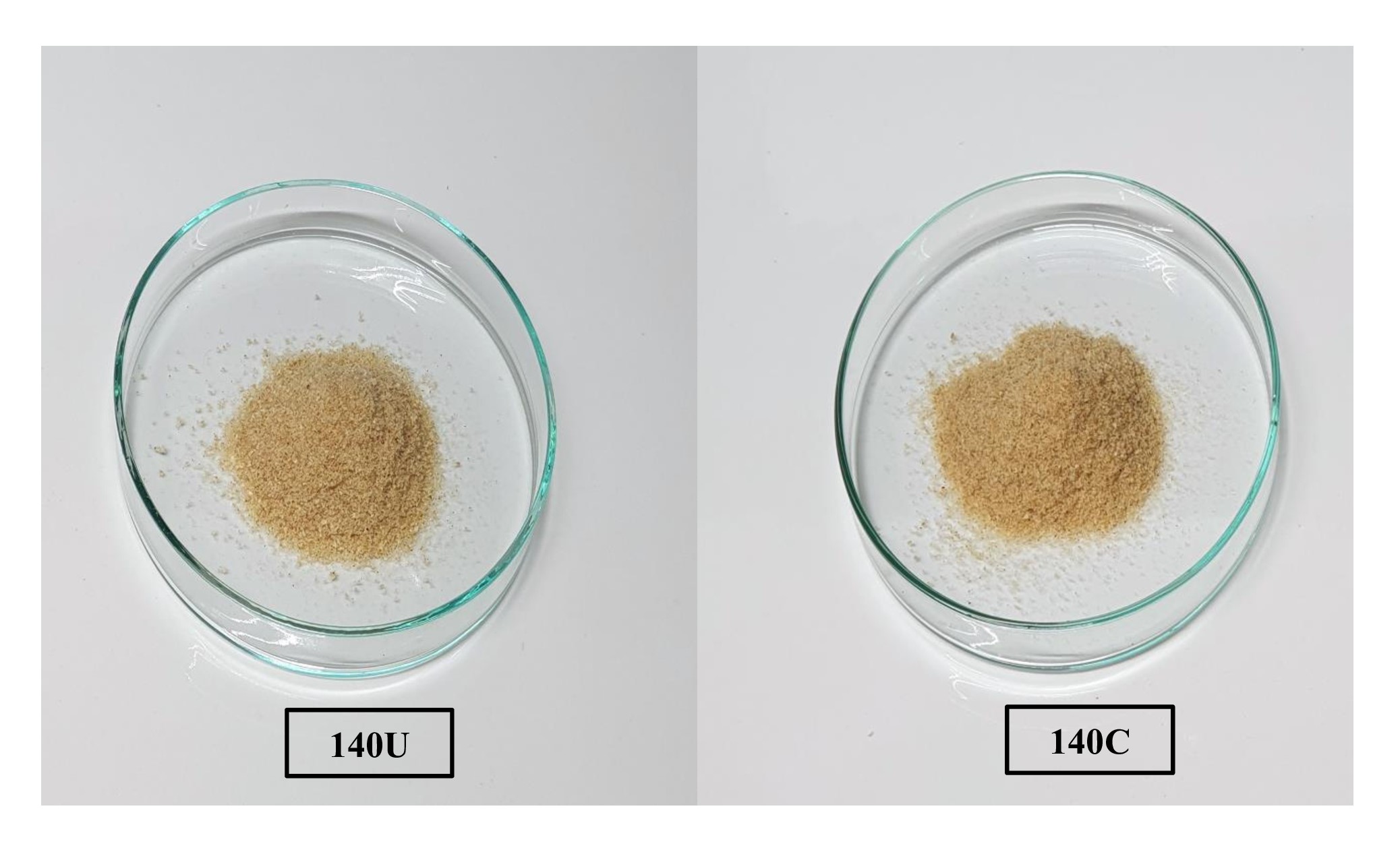

Supplement: Supplementary file 1 [file foods-14-02893-s001.zip › Fig S2.jpg]

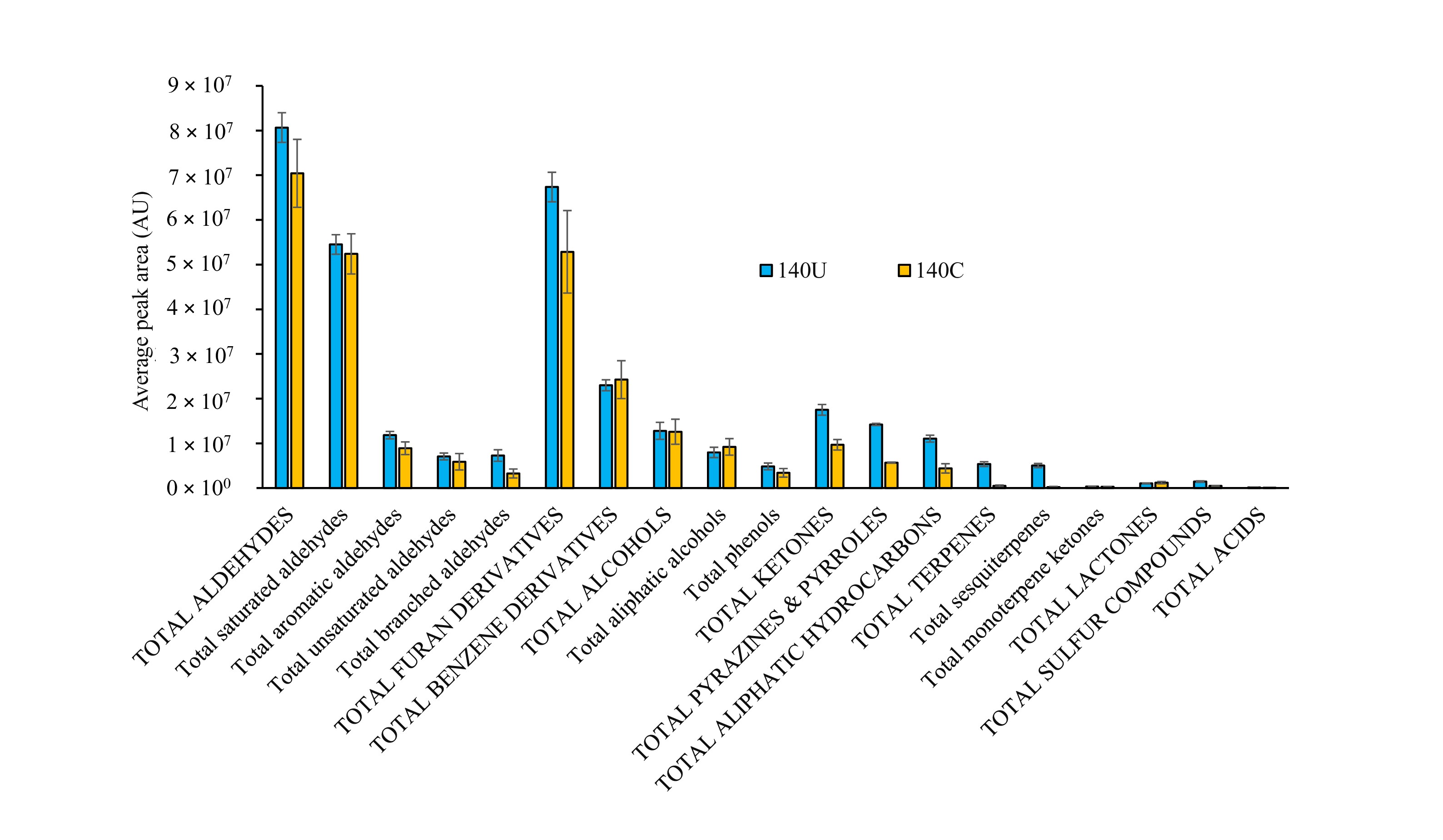

Supplement: Supplementary file 1 [file foods-14-02893-s001.zip › Fig S3.jpg]

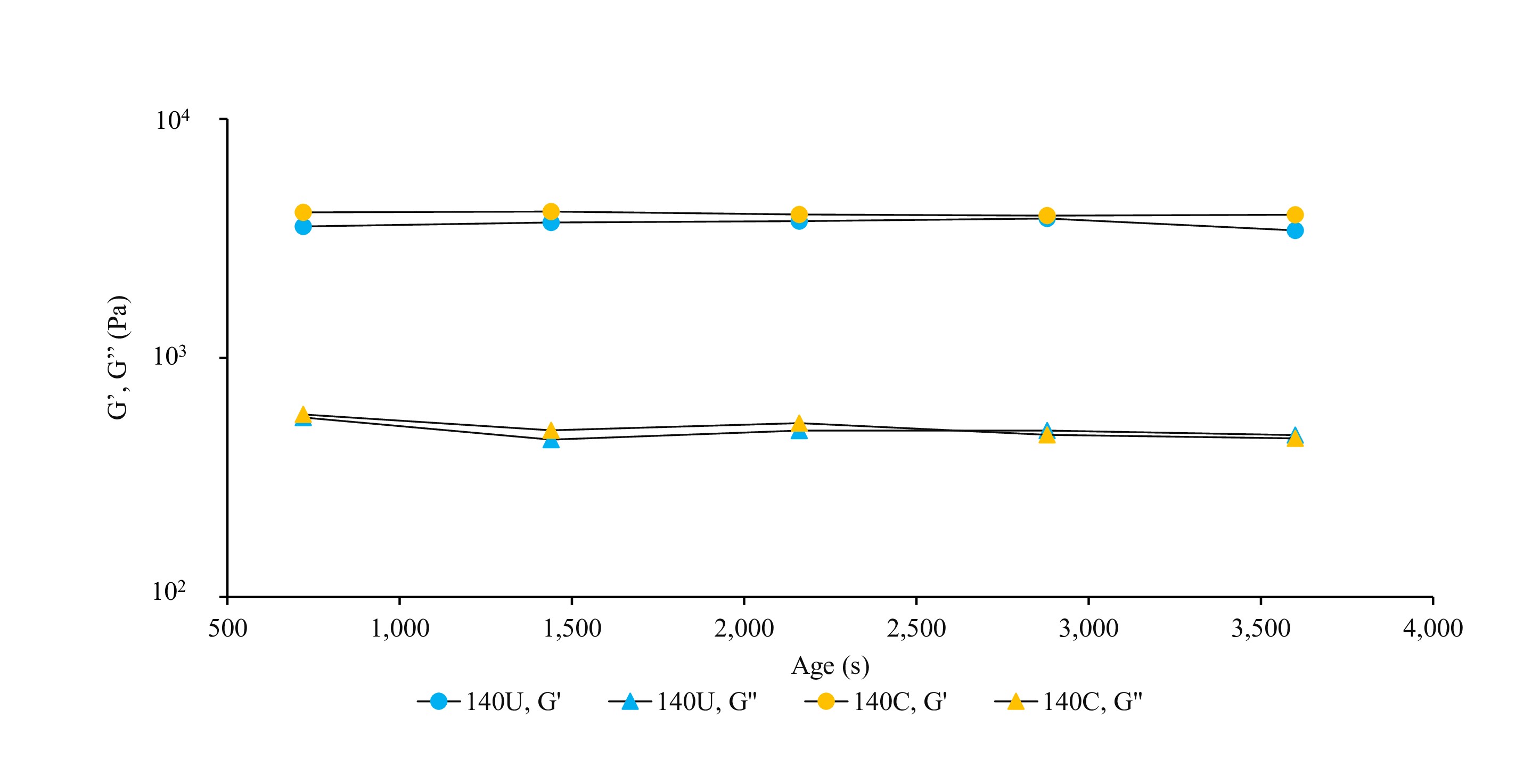

Supplement: Supplementary file 1 [file foods-14-02893-s001.zip › Fig S4.jpg]
